# Overview of the Current Knowledge and Conventional MRI Characteristics of Peri- and Para-Vascular Spaces

**DOI:** 10.3390/brainsci14020138

**Published:** 2024-01-28

**Authors:** Marco Parillo, Federica Vaccarino, Gianfranco Di Gennaro, Sumeet Kumar, Johan Van Goethem, Bruno Beomonte Zobel, Carlo Cosimo Quattrocchi, Paul M. Parizel, Carlo Augusto Mallio

**Affiliations:** 1 Fondazione Policlinico Universitario Campus Bio-Medico, Via Alvaro del Portillo, 200, 00128 Roma, Italy; m.parillo@policlinicocampus.it (M.P.); federica.vaccarino@unicampus.it (F.V.); b.zobel@policlinicocampus.it (B.B.Z.); c.mallio@policlinicocampus.it (C.A.M.); 2 Research Unit of Diagnostic Imaging and Interventional Radiology, Department of Medicine and Surgery, Università Campus Bio-Medico di Roma, Via Alvaro del Portillo, 21, 00128 Roma, Italy; 3 Department of Health Sciences, Chair of Medical Statistics, University of Catanzaro “Magna Græcia”, 88100 Catanzaro, Italy; gianfranco.digennaro@unicz.it; 4 Department of Neuroradiology, National Neuroscience Institute, Singapore 308433, Singapore; sumiluthra@gmail.com; 5 Duke-National University of Singapore Medical School, Singapore 169857, Singapore; 6 Department of Radiology, Antwerp University Hospital, 2650 Edegem, Belgium; johan.vangoethem@uantwerpen.be; 7 Centre for Medical Sciences-CISMed, University of Trento, Via S. Maria Maddalena 1, 38122 Trento, Italy; 8 Royal Perth Hospital & University of Western Australia, Perth, WA 6000, Australia; paul.parizel@health.wa.gov.au; 9 Medical School, University of Western Australia, Perth, WA 6009, Australia

**Keywords:** perivascular spaces, paravascular spaces, Virchow–Robin spaces, PVS, glymphatic system, neurofluids, magnetic resonance imaging, MRI, neuroradiology, brain cysts

## Abstract

Brain spaces around (perivascular spaces) and alongside (paravascular or Virchow–Robin spaces) vessels have gained significant attention in recent years due to the advancements of in vivo imaging tools and to their crucial role in maintaining brain health, contributing to the anatomic foundation of the glymphatic system. In fact, it is widely accepted that peri- and para-vascular spaces function as waste clearance pathways for the brain for materials such as ß-amyloid by allowing exchange between cerebrospinal fluid and interstitial fluid. Visible brain spaces on magnetic resonance imaging are often a normal finding, but they have also been associated with a wide range of neurological and systemic conditions, suggesting their potential as early indicators of intracranial pressure and neurofluid imbalance. Nonetheless, several aspects of these spaces are still controversial. This article offers an overview of the current knowledge and magnetic resonance imaging characteristics of peri- and para-vascular spaces, which can help in daily clinical practice image description and interpretation. This paper is organized into different sections, including the microscopic anatomy of peri- and para-vascular spaces, their associations with pathological and physiological events, and their differential diagnosis.

## 1. Introduction

Perivascular spaces are generally described as fluid-filled areas surrounding arterioles, capillaries, and venules in the brain that allow for the passage of fluid or particles [1]. Durand-Fardel and Pestalozzi first reported the existence of brain perivascular spaces in 1842 and 1849, respectively [1]. Rudolf Virchow and Charles Robin also reported spaces surrounding the perforating vessels in the basal ganglia and hemispheric white matter in postmortem brain specimens visible to the naked eye in 1851 and 1859, respectively [2,3].

Despite being described in the human brain more than 150 years ago, perivascular spaces have gained significant attention in recent years due to advancements of in vivo imaging tools. Moreover, evidence from both clinical and pre-clinical studies indicates that perivascular spaces play a crucial role in maintaining brain health, and their dysfunction is linked to various neurological disorders. The introduction of the glymphatic system hypothesis favored confusion in the literature about the definition and use of terms in describing spaces around and along vessels in the brain’s microanatomy, often used as synonyms (e.g., Virchow–Robin spaces, perivascular spaces (PeVS), and paravascular spaces (PaVS)). It is widely accepted that PaVS function as waste clearance pathways for the brain for materials such as ß-amyloid by allowing an exchange between cerebrospinal fluid (CSF) and interstitial fluid and forming the anatomic foundation of the glymphatic system [4,5]. Although there is no anatomical resemblance, the glymphatic system in the brain is functionally comparable to the peripheral lymphatic system in the body. It probably has an important function in maintaining the brain’s homeostasis by clearing metabolic waste, but it may also be helpful for the distribution of non-waste components, such as glucose [6]. By exploring the compartments of this system, we can better understand the development of common cerebrovascular, neuroinflammatory, and neurodegenerative disorders and the potential role of brain spaces as routes of delivery of therapeutic agents [7] or malignant cells [8]. Nonetheless, several aspects of these spaces have been, and still are, rather controversial. Techniques such as magnetic resonance imaging (MRI) offer a unique opportunity to study the physiology, anatomy, and significance of the brain’s spaces surrounding vessels, and as such, fluid and waste clearance systems.

This article offers an overview of the current knowledge and the MRI characteristics of brain spaces around and along vessels, which can help with daily clinical practice image description and interpretation. This paper is organized into different sections, including the microscopic anatomy of PeVS and PaVS, their possible correlation with pathological and physiological events, and their differential diagnosis.

## 2. Anatomy

**Figure 1:**
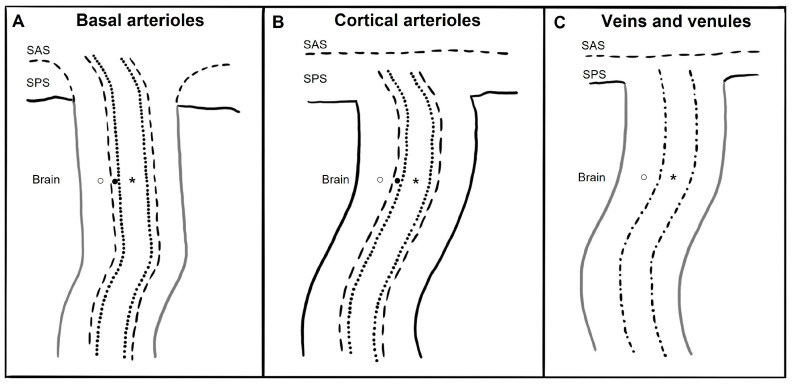
Schematic drawing of paravascular and perivascular spaces currently believed micro-anatomy along and around basal arterioles, cortical perforating arterioles, veins, and venules. SAS: subarachnoid space; SPS: subpial space; ○: paravascular space; ●: perivascular space; *: vessel lumen; dotted line: arterioles endothelium; dash-dot line: vein and venules endothelium; dashed line: meningeal membrane; black line: glia limitans; grey line: meningeal membrane plus glia limitans. (**A**) The brain’s basal arterioles originate from the subarachnoid space and are surrounded by two leptomeningeal membranes. The inner membrane is in close proximity to the arteriolar wall, while the outer membrane is connected to the pia mater, allowing the basal para-arteriolar spaces to have direct communication with the SAS. The perivascular space is another space within the arterial tunica media. (**B**) In cortical arterioles, where there is only one leptomeningeal membrane closely applied to the vessel wall and no outer layer, the para-arteriolar space is believed to communicate with the SPS rather than the SAS. The perivascular space is another space within the arterial tunica media. (**C**) Veins and venules have only one leptomeningeal membrane that is closely attached to the vessel wall without any outer layer, and thus it is believed that the paravenous space communicates with the SPS rather than the SAS. The venous vasculature does not contain perivascular space.

The anatomy of PeVS and PaVS is intricate and remains a matter of debate. In humans, the cortical surface of the brain and the intracranial perforating arterioles are covered by the pia mater [9,10]. Arterioles that traverse the base of the brain and enter the basal ganglia are enveloped by two layers of leptomeninges along their length, forming a space between them that communicates with the subarachnoid space [11,12]. Arterioles in the cortex and venules have a single leptomeningeal membrane that adheres closely to the vessel wall without an outer layer, permitting that the spaces surrounding these vessels communicate with the subpial space directly and, somehow, indirectly with the subarachnoid space [13,14,15]. These spaces can be defined as PaVS and correspond to the “Virchow–Robin spaces”. They are bound internally by the pial sheath and externally by the basement membrane of astrocytes (glia limitans) on the arterial side and the vein wall and the glia limitans on the venous side [16,17]. The glymphatic model suggests that CSF flows through para-arterial spaces, interacts with interstitial fluid and solutes, and then is removed from the brain through para-venous spaces [18]. There are also other spaces within the arterial tunica media, located between the middle layers of the basement membrane of arterial smooth muscle cells [17]. These structures are called PeVS, and they serve as channels for interstitial fluid flow in the opposite direction to that of blood flow (also known as the intramural periarterial drainage pathway) towards the cervical lymph nodes through major cerebral arteries in the neck [16,19]. The venous vasculature does not contain PeVS [17]. In summary, PeVS are spaces around the blood vessels (between the basement membrane of the pial sheath and the basement membrane of the arteries), while PaVS are spaces alongside the blood vessels (outside the vessel wall) (Figure 1).

At the capillary level, the pial sheath’s basement membrane and the glia limitans merge, resulting in a perivascular compartment. It is filled with an extracellular matrix, and it is not connected to the subarachnoid space [20]. At present, it remains unclear whether the paravascular and perivascular compartments in humans are anatomically and functionally contiguous and whether interstitial solute clearance occurs via a periarterial route, a paravenous route, or a combination of both. Additionally, the driving forces that govern these pathways are not fully understood [17]. Further research is needed on the topic to clarify these questions.

## 3. Imaging

Based on the anatomy previously described, PeVS and PaVS are not distinguishable on clinical imaging; we can speculate that what is visible at imaging are more likely the “PaVS” rather than the “PeVS”, but in the absence of sufficient evidence, we use the term PVS to encompass both spaces. Although PVS are located around both arteries and veins, recent studies utilizing 7 Tesla MRI have indicated that the majority of PVS visible with MRI in the centrum semiovale are para-arterial rather than para-venous [11,21,22], but this does not exclude the presence of venular pathology (e.g., venous collagenosis) [23]. The cause of para-venous spaces being less visible at MRI could be related to the smaller size of para-venous spaces and differences in the quantity and/or composition of the para-venous fluid [24]. 

**Figure 2:**
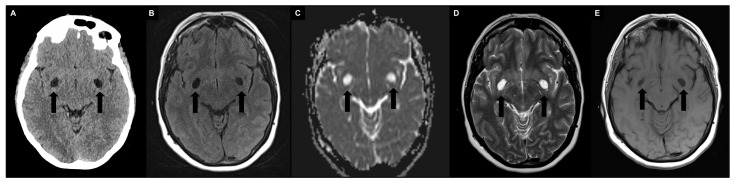
Typical appearance of symmetrically enlarged PVS at the level of the basal ganglia (type I) with densitometric and signal intensity characteristics typically comparable to those of cerebrospinal fluid (black arrows). (**A**) Unenhanced computed tomography shows hypodense PVS. (**B**) A 1.5 T magnetic resonance imaging (MRI) fluid attenuated inversion recovery image shows hypointense PVS. (**C**) A 1.5 T MRI apparent diffusion coefficient image shows no diffusion restriction. (**D**) A 1.5 T MRI T2-weighted turbo spin echo image shows hyperintense PVS with a microscopic central vessel inside, known as the “vessel sign”. (**E**) A 1.5 T MRI T1-weighted spin echo image shows hypointense PVS.

PVS may contain proteinaceous material, including extracellular matrix, fibrin/fibrinogen, and hemosiderin deposits, in addition to interstitial fluid [25,26]. However, it is still unclear how these deposits affect the signal and visibility of PVS on MRI in vivo [27,28]. Hence, the densitometric and signal intensity characteristics of PVS are typically comparable to those of CSF (Figure 2) [24]. 

PVS appear on computed tomography (CT) scans as well-circumscribed fluid-density spaces without contrast enhancement or calcifications. On MRI, PVS display hypointensity on T1-weigthed and fluid-attenuated inversion recovery (FLAIR) images, as well as hyperintensity on T2-weighted images, with no signs of contrast enhancement or mass effect; the signal of the surrounding brain is typically normal. Since PVS are communicating compartments, they do not exhibit restricted diffusion on diffusion-weighted imaging (DWI) [29]. A high-resolution MRI can reveal a microscopic central vessel within the PVS, known as the “vessel sign” [30]. Indeed, MRI has greater sensitivity in detecting PVS than CT, especially using T2-weighted and cisternographic sequences [29], and contrast agent administration is not required (avoiding the problems associated with gadolinium deposits [31,32]). Regarding morphology, when the penetrating artery is parallel to the imaging plane, PVS seems to have a striped appearance, whereas when they are perpendicular, PVS appear rounded or ovoid [29]. In terms of location, PVS are usually observed symmetrically in certain areas of the brain, such as the basal ganglia (including the lentiform nucleus, along the anterior commissure, and in the internal and external capsules), located just above the anterior perforated substance, and the centrum semiovale, running towards the lateral ventricles from the external aspect of the white matter with the highest concentration (PVS-to-white-matter ratio) in the subinsular white matter [33]. They are also found in the hippocampus, midbrain, pons, and, in some cases, in cerebellar white matter [1]. PVS are commonly categorized into three types based on their location. Type I PVS, which are frequently observed with MRI, are located along the lenticulostriate arteries that enter the basal ganglia through the anterior perforated substance. Type II PVS can be seen along the course of the perforating medullary arteries as they enter the cortical gray matter over the high convexities and extend into the white matter. Type III PVS are located in the midbrain and follow the path of the penetrating branches from the posterior cerebral artery [34,35]. Regarding size, PVS typically have a cross-sectional diameter of less than 2 mm on MRI. When the diameter is between 3–5 and 15 mm, PVS are considered enlarged; those measuring 15 mm or more are defined as giant/tumefactive PVS, also known as “cavernous dilatation” or Poirier’s Type IIIb “expanding lacunae” [36,37,38,39]. 

**Figure 3:**
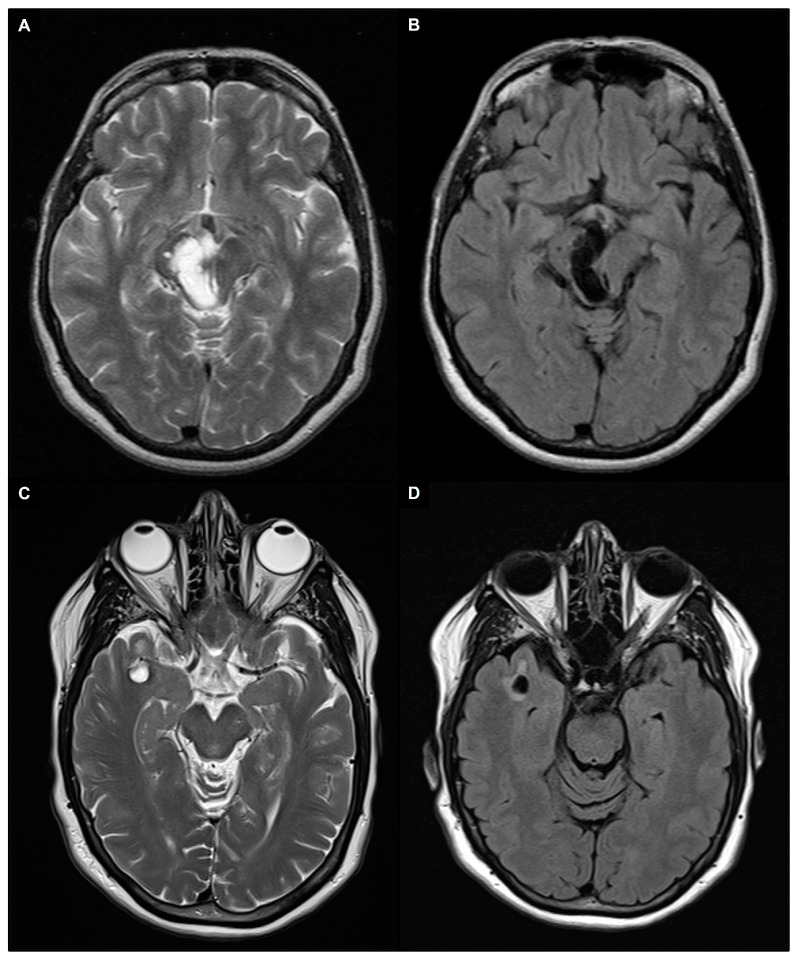
A 1.5 T magnetic resonance imaging appearance of giant PVS in T2 turbo spin echo images (**A**,**C**) and fluid attenuated inversion recovery (FLAIR) images (**B**,**D**). (**A**,**B**) Patient with giant PVS in the right mesencephalothalamic region (type III) that cause a mass effect on the aqueduct of Sylvius. (**C**,**D**) Patient with a right anterior temporal lobe enlarged perivascular space (type IV) characterized by an elevated perilesional FLAIR signal with no mass effect, which indicates gliosis.

Enlarged and giant PVS may show surrounding T2/FLAIR signal abnormalities, indicating gliosis [40]. Giant PVS are most commonly found in the mesencephalothalamic region (type III location), where they can cause a mass effect on the aqueduct of Sylvius and finally lead to obstructive hydrocephalus (Figure 3A,B) [41,42,43]. While headaches are the most common symptom, other non-specific neurological symptoms such as memory loss, seizures, dizziness, syncope, visual, balance, or concentration impairment may also be present. In addition, even very large PVS can be completely asymptomatic [44,45]. A specific type of tumefactive PVS, referred to as “type IV”, has been identified in the subcortical white matter of the anterior superior temporal lobe. These giant PVS typically have an elevated perilesional FLAIR signal with no mass effect that indicates gliosis and are commonly related to a branch of the middle cerebral artery and a focal region of cortical absence or thinning (Figure 3C,D) [46,47].

Diffusion MRI is a non-invasive imaging technique that detects the movement of water molecules by utilizing magnetic field gradients in various directions. By applying these gradients, diffusion MRI can examine the local micro-environment in the brain, offering a unique ability to encode directional information and distinguish between different microstructural tissue features, thanks to the structural barriers imposed by cell membranes. While conventional use of diffusion MRI focuses on modeling white matter structure, it can also be applied to assess fluid dynamics in PVS [24]. The technique known as diffusion tensor imaging analysis along the perivascular space (DTI-ALPS) involves isolating diffusivity along the PVS, projection fibers, and association fibers, leading to the calculation of the DTI-ALPS index, with an elevated index indicating increased diffusivity along the PVS. Notably, the DTI-ALPS index mainly provides information on the diffusion of interstitial fluid within the spaces that surround the medullary veins. To address non-specificity in DTI interpretations, a multi-compartment modeling approach is employed. This involves acquiring diffusion-weighted images at various b-values or gradient strengths to probe different tissue compartments characterized by distinct diffusion profiles [48]. Examining the diffusion of interstitial fluid within the PVS provides valuable insights into the PVS microstructure, including pathological enlargement and function, such as disruptions in glymphatic flow due to debris presence. The diffusion process extends beyond the PVS, reaching the extracellular space of the surrounding parenchyma. This broader perspective offers information on the effectiveness of glymphatic influx and efflux mechanisms, shedding light on overall glymphatic system dynamics [24].

## 4. Quantification

Precise measurement of the enlarged PVS is important, especially for research purposes, so that their potential correlations can be studied. Traditionally, visual scores have been used for quantification, and more recently, computational image analysis methods have been implemented.

### 4.1. Visual Scoring

**Figure 4:**
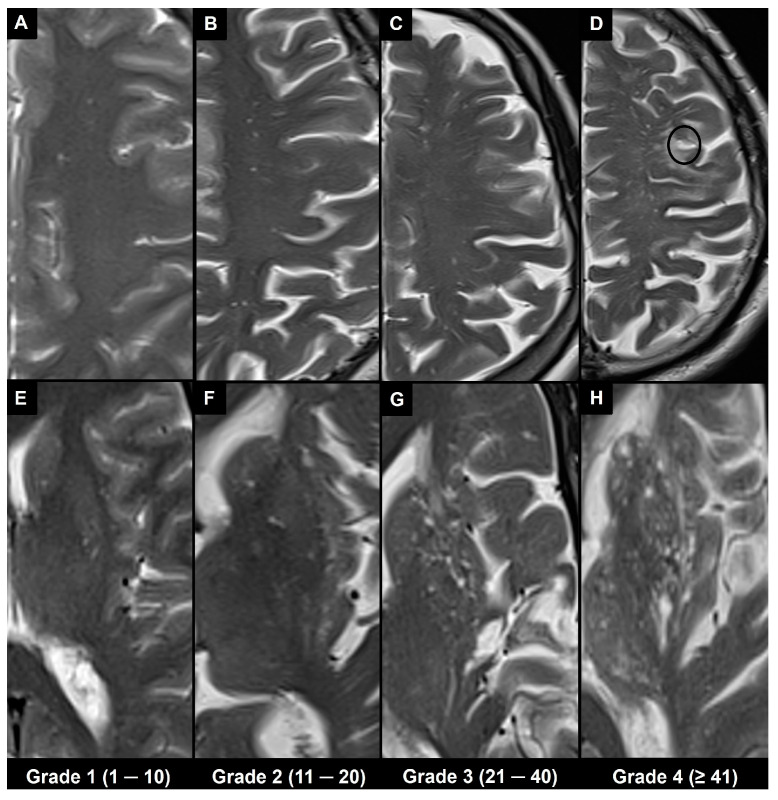
The 1.5 T magnetic resonance imaging T2 turbo spin echo images in different patients show the Potter scoring system based on counting visible PVS. Each hemisphere is scored separately for PVS, and the higher score of the two is used, as indicated at the bottom. (**A**–**D**) Axial slices at centrum semiovale level (type II). (**E**–**H**) Axial slices at basal ganglia level (type I), where H corresponds to the “état criblé” or “status cribrosum” described by Durand-Fardel in the basal ganglia. Note also the presence of a lacune in (**D**) (black circle) and that PVS in the centrum semiovale can be observed as they approach the cortex; their dilation increases as they reach the inner edge of the cortex; however, they cannot be seen as they pass through the cortex.

Different types of visual rating systems have been implemented, including the Potter scoring system [49]. This system categorizes PVS into five grades based on their number in the basal ganglia and centrum semiovale (grade 0 = none; grade 1 = 1–10; grade 2 = 11–20; grade 3 = 21–40; grade 4 ≥ 41 corresponding to the “état criblé” or “status cribrosum” described by Durand-Fardel in the basal ganglia [1]). Each hemisphere is scored separately, and the higher score of the two is used (Figure 4). 

For the midbrain, a score of 0 or 1 is used based on the absence or presence of PVS [49]. The grading of the hippocampal region based on the number of PVS was performed by the Chinese IntraCranial AtheroSclerosis study group, dividing PVS into three grades (grade 1 ≤ 5; grade 2 = 6–10; grade 3 ≥ 11) [50].

Other assessment methods incorporate the size of the PVS. Heier categorized PVS based on their diameter (mild grade 1 < 2 mm, moderate grade 2 = 2–3 mm, and severe grade 3 > 3 mm) [34]. Another study evaluated PVS by grading the presence or absence of PVS with a cross-sectional diameter > 3 mm, referred to as an enlarged PVS [51].

Visual rating scales have multiple benefits, as they are simple to understand and apply, can be implemented on both T2-weighted and T1-weighted images, do not require specialized equipment or isotropic acquisition, and can be applied directly to standard clinical images without the need for specialized workstations or software [24]. On the other hand, visual rating scales have some drawbacks, such as limited sensitivity, inter- and intra-reader variability, and a restricted number of PVS characteristics that can be determined. Additionally, the visual evaluation of small PVS can be influenced by technical parameters and can be challenging and time-consuming, hindering the application of these scales to large datasets [52].

### 4.2. Automatic and Semi-Automatic Segmentation and Morphometry

Advances in technology have led to the emergence of various automatic and semi-automatic methods for segmenting and quantifying PVS. Once the PVS are segmented, various metrics and morphological features can be computed from segmentation masks [24,52]. The PVS volume is an indicator of the amount of fluid within the PVS [33]. The mean cross-sectional diameter can be utilized to differentiate PVS from other cerebral small vessel disease-related lesions [53]. Linearity represents the similarity of a particular PVS cluster to the tubular morphology [54], while solidity reflects the shape complexity of PVS, where a lower solidity indicates a more tortuous and less compact shape [24]. With the increased availability of large neuroimaging datasets and high-resolution images in recent years, data-driven techniques like machine learning and deep learning have become popular methods for PVS segmentation tasks [55,56,57,58]. Sepehrband et al. developed a multimodal technique called Enhanced PVS Contrast to enhance the visibility of PVS on MRI, combining T1- and T2-weighted images and applying a filtering algorithm to remove “non-structured high frequency spatial noise”. The resulting image significantly improves the visibility of PVS, making it suitable for the automatic quantification of PVS [59]. Gonzàlez-Castro et al. introduced an entirely automated approach employing a support vector machine (SVM) for categorizing the burden of PVS on T2-weighted images in the basal ganglia area as either low or high. The efficacy of three distinct descriptor types was evaluated, and the SVM classifier demonstrated superior accuracy with the “bag of visual words”-based descriptor (81.2%). The concordance between the classifier and two observers was notably strong (κ > 0.61) [60]. Dubost et al. applied convolutional neural network regression as a deep learning algorithm to assess and quantify PVS in various brain regions such as the midbrain, hippocampi, basal ganglia, and centrum semiovale. The comparison between visually assessed scores and automated scores revealed a high level of agreement across all four regions, with intraclass correlation coefficients ranging between 0.75 and 0.88 [61]. In a separate investigation, Dubost et al. suggested the utilization of a convolutional network regression approach to measure the degree of enlarged PVS in the basal ganglia based on a 3D brain MRI. The intraclass correlation coefficient between this scoring method and the visual assessments by an expert yielded a value of 0.74 [62]. Additionally, they introduced a weakly supervised detection method employing neural networks. This method calculated attention maps that revealed the locations of brain lesions across four brain regions, achieving outcomes that closely aligned with human intrarater agreement in each respective region [63]. Williamson et al. designed a 3D network to assess the severity of enlarged PVS in the basal ganglia using clinical-grade imaging in an acute stroke cohort of patients, achieving an accuracy of 89.7% [64]. These automated segmentation techniques will increasingly be utilized in clinical settings, offering valuable information on the progression of PVS in relation to aging and medical conditions, possibly overcoming the limitations of visual scores with results close to those from a trained observer.

## 5. Associations

In the past, the visualization of PVS was considered an indicator of neurological disease. However, with advances in MRI technology, more PVS can now be detected compared to older studies. While it may be challenging to visualize non-enlarged PVS using a 1.5 Tesla MRI, higher magnetic field strength (≥3 Tesla) allows for adequate resolution and signal-to-noise ratio to observe PVS morphology in nearly all individuals, including healthy adolescents and young adults [33,65]. Therefore, the presence of MRI-visible PVS does not necessarily indicate an underlying pathology. 

### 5.1. Physiological

It is widely known that the visibility of PVS increases with age, especially in the basal ganglia, centrum semiovale, and hippocampus [66]. A recent study documented low PVS volumes in the basal ganglia and centrum semiovale in healthy subjects up to the age of 35 despite high Potter visual scores in the white matter of young subjects; also, it was suggested that in the basal ganglia, a Potter grade 1 and a Potter grade 3 could be considered physiological until the age of 50 and from the age of 70 onward, respectively, while in the centrum semiovale, a Potter grade 4 could be considered physiological from the age of 50 [67]. A higher PVS burden has been associated with the male sex, and it is also seen in healthy adolescents [33,65]. A circadian dynamic nature of the amount of MRI-visible PVS (higher PVS burden is visible on MRI at later times of the day) with a predominantly asymmetric distribution has been suggested. This could be associated with circadian fluctuations in respiration or/and blood pressure (which are known regulators of perivascular flow) or may be linked to the circadian control of flow through aquaporin-4 (a water channel that facilitates fluid transport from the PVS to the cerebral parenchyma) [33]. Furthermore, cosmonauts and astronauts have exhibited augmented PVS burden in both the basal ganglia and white matter after their space travel. Extended exposure to microgravity has the potential to modify the circulation between CSF and interstitial fluid in PVS. This alteration could potentially hinder cerebral drainage systems such as the glymphatic pathway, emphasizing the significance of maintaining gravitational influences for proper brain fluid homeostasis [68].

### 5.2. Pathological

To date, several systemic and neurological diseases, including neurovascular, neurodegenerative, and neuroinflammatory conditions, have been associated with a higher visibility of PVS on MRI.

A recent review proposed four major etiologies, which may partly overlap, to explain the high burden of PVS in these conditions. These include (1) the impairment of interstitial fluid circulation, which may be caused by protein aggregate deposition, arterial/venous pathology, sleep disturbances, and disruption of cerebrospinal fluid CSF—interstitial fluid efflux routes; (2) the elongation/tortuosity of arteries in a spiral shape; (3) the paravascular myelin loss and/or brain atrophy; and (4) the accumulation of immune cells in the PVS [69].

It is widely known that the visibility of PVS in the basal ganglia (but not in the centrum semiovale) increases with hypertension [66], and hypertension was found to be related to a low DTI-ALPS index [70]. Historically, MRI biomarkers of cerebral small vessel disease have included enlarged PVS along with other indicators such as recent small subcortical infarcts, lacunes, white matter hyperintensities (WMH) of presumed vascular origin, cerebral microbleeds, and brain atrophy, which are often present in association with stroke or cognitive decline. PVS are related both to lacunes and to microbleeds, having a positive correlation with WMH [29,66]. Moreover, patients who had a high number of visible PVS demonstrated a high incidence of recurrent ischemic stroke [71]. The DTI-ALPS technique has been widely employed in studies involving aging and small vessel disease cohorts. In all instances, a consistent finding emerged, indicating an inverse correlation between the load of WMH and the diffusion of venous interstitial fluid in the PVS [48]. Research using the DTI-ALPS approach additionally demonstrated diminished fluid dynamics in the venous PVS following occurrences of ischemic stroke [72] and intracerebral hemorrhage [73]. PVS are frequently observed on MRI scans of patients with cerebral autosomal dominant arteriopathy with subcortical infarcts and leukoencephalopathy (CADASIL), distributed across the entire brain, particularly in the temporal lobes and subinsular areas [74].

Alzheimer’s disease, characterized by an abnormal accumulation of A*β* in the brain and decreased functional connectivity [75], has been found to be significantly correlated with a larger PVS volume and count as well as higher visual severity ratings in the subcortical white matter [15,76,77,78]. Studies employing DTI-ALPS have observed a decline in the ALPS index along the medullary vessels in individuals diagnosed with Alzheimer’s disease [79]. In patients with mild cognitive impairment, the PVS volume fraction was found to be higher in the centrum semiovale of the white matter in females only, while it was lower in the anterosuperior medial temporal lobe [80]. However, some studies have not found any significant associations between the burden of MRI-visible PVS and cognitive function [81]. The deposition of A*β* in cerebral arteries is a characteristic of cerebral amyloid angiopathy, which is linked to a high burden of PVS in white matter [82,83], especially in patients with recurrent intracerebral hemorrhage [84].

Parkinson’s disease, characterized by the presence of Lewy bodies from the abnormal aggregation of α-synuclein protein, is linked to a greater PVS load in the basal ganglia and in certain regions of the subcortical white matter [85,86]. Individuals with Parkinson’s disease who had mild cognitive impairment and those with Parkinson’s disease-related dementia exhibited notably lower DTI-ALPS indices when compared to the control group. However, this trend was not observed in Parkinson’s disease patients with normal cognitive function [87].

Enlarged PVS in the white matter have been observed in individuals with multiple sclerosis, a neuroinflammatory condition that is distinguished by demyelinating lesions and accelerated brain volume loss [88,89,90,91]. PVS were also visible in the centrum semiovale of patients with systemic lupus erythematosus, suggesting a link with systemic disease activity [92]. It has also been observed that convexity visible PVS may be an early finding in myotonic dystrophy patients, preceding the appearance of WMH [93]. Furthermore, unusually large PVS in the centrum semiovale are seen in children and adolescents with autism [94].

These data suggest that enlargement of PVS at the level of the basal ganglia is more typical of hypertension, lacunar stroke, and Parkinson’s disease, while greater white matter involvement is more frequently associated with dementia, cerebral amyloid angiopathy, multiple sclerosis, systemic lupus erythematosus, myotonic dystrophy, and autistic spectrum disorders.

The number of PVS visible on MRI and the enlargement of PVS have been linked to various other pathological conditions such as high body mass index [33] and a high amount of visceral fat [95], sleep dysfunction [96] and obstructive sleep apnea [97], spaceflight-associated neuro-ocular syndrome [68], amyotrophic lateral sclerosis [98], mutations in type IV collagen [99], Sener syndrome [100], pediatric idiopathic generalized epilepsy [101], depression [102], and traumatic brain injury [103].

These observations suggest that PVS may play an important role in the pathophysiology of a number of diseases as an indicator of intracranial pressure and neurofluid imbalance. Future research is needed to better understand the etiology, clinical significance, and potential therapeutic target of PVS enlargement in various diseases. Table 1 summarizes the main associations between visible PVS and PVS burden on brain MRI and physiological or pathological conditions.

## 6. Differential Diagnosis

**Figure 5:**
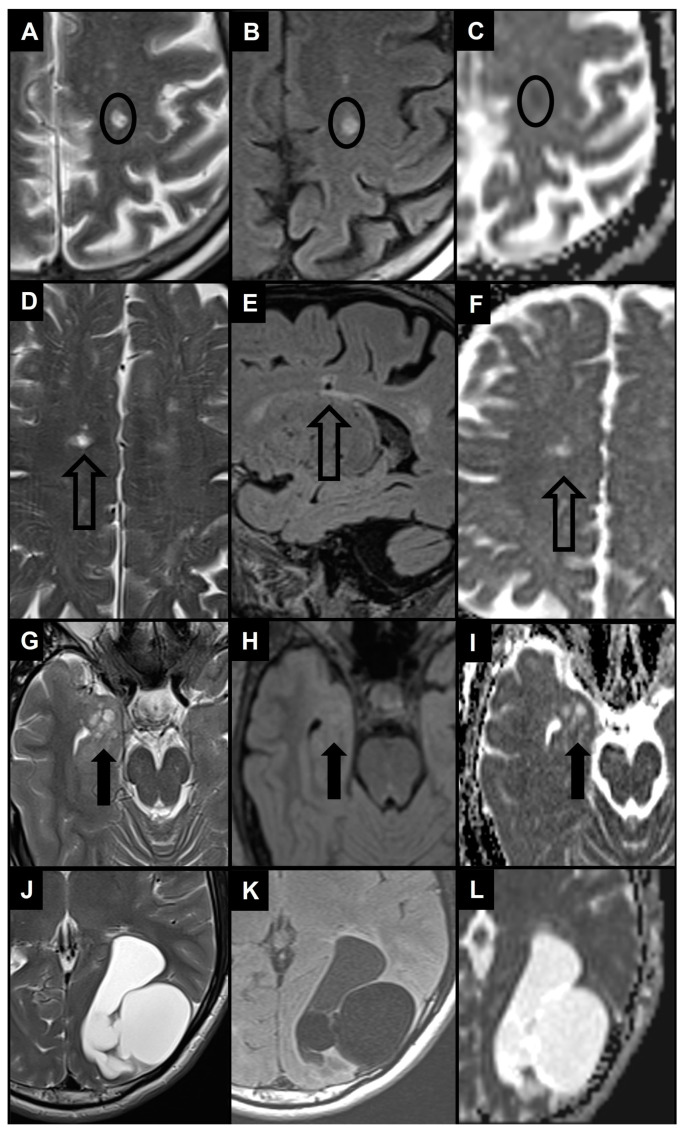
The 1.5 T magnetic resonance images show the main differential diagnosis for enlarged PVS in the context of cerebral small vessel disease (**A**–**F**) and 2 examples of differential diagnosis for giant PVS (**G**–**L**) using T2 turbo spin echo images (**A**,**D**,**G**,**J**), FLAIR images (**B**,**E**,**H**,**K**) and apparent diffusion coefficient images (**C**,**F**,**I**,**L**). (**A**,**B**) Patient with a small area of altered signal in the left centrum semiovale (black circle) characterized by hyperintensity in T2 and FLAIR images and diffusion restriction, therefore compatible with a recent subcortical infarction. (**D–F**) Patient with a small area of altered signal in the right centrum semiovale (empty black arrow) characterized by hyperintensity in the T2 image, hyperintense rim in the FLAIR image, and no diffusion restriction, therefore compatible with a lacune. Note also the white matter hyperintensities in centrum semiovale bilaterally. (**G**–**I**) Patient with a dysembryoplastic neuroepithelial tumor in the right medial temporal lobe (thick black arrow), with a distinct “bubbly” cystic appearance associated with T2/FLAIR signal abnormality and no diffusion restriction. (**J**–**L**) Patient with a left parieto-occipital neurenteric cyst characterized by a slightly hyperintense content in FLAIR images and no diffusion restriction.

Enlarged PVS (diameter between 3–5 and 15 mm) most frequently need to be differentiated from lacunes and WMH as they are similar in signal characteristics and they often coexist in the context of cerebral small vessel disease (Figure 5A–F) [29,104]. When PVS appear notably enlarged (e.g., giant PVS with diameter ≥ 15 mm), causing mass effect, and presenting cystic configurations, they must be distinguished from other cystic pathologies of possible congenital, vascular, inflammatory, neoplastic, or hereditary nature (Figure 5G–L) [105,106]. In clinical practice, by bringing together information from conventional MRI sequences and from the patient’s clinical—anamnestic picture, a very likely diagnosis can be reached. In doubtful cases, follow-up of the lesion itself helps to distinguish a benign from a malignant cyst.

Table 2 summarizes the main differential diagnosis.

### 6.1. Recent Small Subcortical Infarct

It is an infarct that occurred within the territory of a perforating arteriole in a time span of a few weeks. It typically has a maximum diameter of ≤20 mm in the axial plane, exhibits restricted diffusion on DWI, and increased signal intensity on FLAIR and T2-weighted sequences [29].

### 6.2. Lacune of Presumed Vascular Origin

It is characterized by a subcortical, round, or ovoid cavity filled with fluid that typically ranges from 3 mm to 15 mm in diameter. It is commonly related to a prior small subcortical infarct or hemorrhage that occurred within the territory of a perforating arteriole [29]. In FLAIR images, lacunes typically exhibit a central hypointensity resembling that of CSF, surrounded by a hyperintense rim. Nevertheless, the presence of a rim is not always detectable, and PVS may also have a hyperintense rim when passing through an area of WMH. When the suppression of fluid signal in the central cavity is not achieved with FLAIR imaging, it may have a clear CSF-like intensity on T1-weighted and T2-weighted sequences. When lacunes are numerous, the radiological picture is described as “état lacunaire”, a possible substrate for multi-infarct vascular dementia [107]. Asymmetrical lesions with a wedge-shaped appearance and a diameter >3 mm are often identified as lacunes rather than PVS [29]. Lacunes are also more prevalent in the upper two-thirds of the basal ganglia, while basal ganglia PVS are more often located in the lower third, near the anterior commissure [108].

### 6.3. WMH

WMH of presumed vascular origin are bright on FLAIR and T2-weighted images and may appear as iso- or hypointense on T1-weighted sequences, without cavitation (different signal from CSF) [29]. The distribution patterns of WMH and PVS are similar, as they tend to be symmetric. However, WMH are often located in the periventricular region and follow the contour of the lateral ventricles, while PVS are typically not present in this area. Moreover, PVS in the white matter generally start from the lower edge of the cortical layer and progress towards the lateral ventricles, following the path of penetrating vessels, while deep WMH are often not directly in contact with the cortical layer [37].

### 6.4. Benign Intracranial Cysts

There are several types of noncancerous cystic masses resulting from congenital abnormalities with different embryological origins [109]. Choroidal fissure cysts refer to non-cancerous cysts that develop within the choroidal fissure [110]; hippocampal sulcus remnant cysts resemble a “string of beads” between the cornu ammonis and dentate gyrus [111]. The differentiation with PVS is based on their specific location within the brain. Neuroglial cysts do not have any communication with the ventricular system or arachnoid space and are distinct from PVS as they are solitary lesions, while arachnoid cysts are extra-axial CSF fluid collections commonly observed in the perisellar cysterns and middle cranial fossa; thus, their location and solitary nature are the main distinguishing factors separating them from clusters of PVS. Neurenteric cysts, due to their proteinaceous contents, are often hyperintense on FLAIR and T1-weighted images [40].

### 6.5. Neoplastic Intracranial Cysts

It is possible to misclassify enlarged PVS as multinodular and vacuolating neuronal tumors and dysembryoplastic neuroepithelial tumors. Multinodular and vacuolating neuronal tumors are a type of benign, mixed glial neuronal lesion associated with seizures. They are a cluster of small, cystic, and nodular lesions located in the subcortical region in the deep cortical ribbon or in the convexities white matter. They show T2/FLAIR hyperintensity and rarely exhibit progression or contrast enhancement [40,112]. Dysembryoplastic neuroepithelial tumors are WHO grade I tumors that are typically found in the medial temporal lobes and cause complex partial seizures. They are generally benign and slow-growing, with a distinct “bubbly” cystic appearance associated with T2/FLAIR signal abnormality and faint contrast enhancement [40,113]. Moreover, different types of low-grade cystic neoplasms can imitate the appearance of enlarged PVS. Cystic brain tumors like gangliogliomas, pleomorphic xanthroastrocytomas, and pilocytic astrocytomas may show contrast enhancement (in relation to the presence of solid tissue), may have restricted or facilitated diffusion, may show surrounding edema, and may have different contents than CSF in FLAIR sequences [35,44]. In summary, apart from the typical appearance of cysts filled with CSF having various sizes, shapes, and numbers, PVS can be differentiated from cystic neoplasms based on the absence of contrast enhancement and their consistent appearance over time.

### 6.6. Vascular and Inflammatory Cysts

Porencephalic cysts, secondary to a focal cerebral injury in early pregnancy, may occur in the periventricular white matter [114]. These cysts have unusual T2/FLAIR signals, connections with the ventricular system, and/or subarachnoid space, which distinguishes them from enlarged PVS. Cystic periventricular leukomalacia, resulting from hypoxic ischemic events during pre- or peri-natal age, is commonly observed in premature infants with cerebral palsy. Loss of periventricular white matter, mostly in periatrial regions, is evident with focal ventricular enlargement near abnormal hyperintense white matter in T2/FLAIR images. The damage tends to be symmetric, and thinning of the corpus callosum may also occur. Previous hemorrhage may also cause hypointensity in SWI [35,115]. 

The periventricular and juxtacortical white matter can show ovoid lesions filled with CSF due to chronic multiple sclerosis [116]. The surrounding T2/FLAIR signal is hyperintense, and the clinical history helps distinguish multiple sclerosis plaques from PVS as well as other vascular and inflammatory insults.

### 6.7. Infectious Cysts

Multiple cystic lesions in the brain can be caused by rare infectious processes. The most prevalent parasitic infection in the CNS and a major cause of acquired epilepsy worldwide is neurocysticercosis, caused by *Taenia solium*. The gray-white matter junction, basal ganglia, brainstem, cerebellum, subarachnoid space, ventricles, or spinal cord may contain fluid-filled oval cysts with an internal scolex (cysticerci). The MRI appearance of neurocysticercosis depends on the infection’s evolution stage. In particular, during the initial vesicular stage, the lesion appears like an enlarged PVS, but the presence of an eccentric scolex, hyperintense to CSF, may help in diagnosis [35,117]. Opportunistic fungal infection caused by *Cryptococcus neoformans*, known as cryptococcosis, should be suspected in individuals with enlarged PVS who are immunocompromised, including those infected with human immunodeficiency virus (HIV). Cryptococcosis typically begins as basal meningitis, leading to hydrocephalus. As the infection spreads along vessels, the PVS may become enlarged with mucoid or gelatinous material from the organism’s capsule. T2-weighted and FLAIR images can differentiate these hyperintense lesions from normal PVS; contrast enhancement is rare and DWI shows restricted diffusion cyst content [118,119]. Neurotoxoplasmosis is another opportunistic infection caused by the parasite *Toxoplasma gondii*; the disease is transmitted via exposure to cat feces, with cats serving as the primary host. MRI shows multiple lesions that may exhibit intrinsic T1 shortening and/or susceptibility and may enhance in a ring-like pattern (the so-called “target sign” in the basal ganglia and corticomedullary junction) [120,121].

### 6.8. Inherited Cysts

Among hereditary disorders, mucopolysaccharidoses should be considered in the differential diagnosis of enlarged PVS. They are a group of metabolic disorders identified by a lack of enzymes causing an inability to break down glycosaminoglycan, leading to toxic intracellular substrate accumulation. Enlarged PVS are a common occurrence due to the accumulation of glycosaminoglycan, with a cribriform appearance on T1-weighted images of the basal ganglia, corpus callosum, and white matter. The PVS appear isointense to CSF on both T2-weighted and FLAIR images, but there may be an increased signal intensity in the surrounding white matter, which could signify dys- or demyelination, edema, or gliosis. The clinical picture is characterized by macrocephaly, musculoskeletal deformities, and mental and motor impairment [35,122,123].

## 7. Conclusions

The imaging of PVS has become an important target of routine brain MRI interpretation, also related to the increased diagnostic capacity of current MRI equipment and sequences. Visible PVS on MRI have been associated with a wide range of neurological and systemic conditions, although this finding is currently not specific. Radiologists play a crucial role in identifying and characterizing PVS as they may provide important diagnostic and prognostic information considering their potential as an early indicator of vascular dysfunction and neurodegenerative/neuroinflammatory disorders. Thus, it is important for brain specialists to be familiar with the terminology and classification systems for PVS, as well as their normal and abnormal appearances, clinical meaning, and differential diagnosis. Further research is needed to better understand the possible underlying pathophysiology and clinical significance of PVS and how imaging features can aid in measuring their compliance and detecting individual vulnerabilities.

## Figures and Tables

**Table 1:** Main location of visible PVS and PVS burden on brain magnetic resonance imaging in physiological and pathological conditions.

	Condition	Main PVS Burden Location
Physiological	Aging [66,67]	Basal ganglia and centrum semiovale
	Male sex [33]	Centrum semiovale
	Later time of day [33]	Basal ganglia and centrum semiovale
	Spaceflights [68]	Basal ganglia and centrum semiovale
Pathological	Hypertension [66]	Basal ganglia
	Cerebral small vessel disease [29,66]	Basal ganglia
	Alzheimer’s disease [15,76,77,78]	Centrum semiovale
	Cerebral amyloid angiopathy [82,83]	Centrum semiovale
	Parkinson’s disease [85,86]	Basal ganglia
	Multiple sclerosis [88,89,90,91]	Centrum semiovale
	Spaceflight-associated neuro-ocular syndrome [68]	Centrum semiovale
	High BMI/visceral fat [33]	Basal Ganglia
	Sleep dysfunction [96]	Centrum semiovale
	Obstructive sleep apnea [97]	Basal ganglia and centrum semiovale
	Myotonic dystrophy [93]	Centrum semiovale
	Amyotrophic lateral sclerosis [98]	Basal ganglia and centrum semiovale
	Systemic lupus erythematosus [92]	Centrum semiovale
	CADASIL [74]	Centrum semiovale
	Mutations in type IV collagen [99]	Basal ganglia and centrum semiovale
	Sener syndrome [100]	Centrum semiovale
	Pediatric idiopathic generalized epilepsy [101]	Centrum semiovale
	Autism spectrum disorders [94]	Centrum semiovale
	Depression [102]	Centrum semiovale

BMI: body mass index; CADASIL: cerebral autosomal dominant arteriopathy with subcortical infarcts and leukoencephalopathy.

**Table 2:** Main differential diagnostic considerations and imaging features for enlarged PVS and giant PVS on brain magnetic resonance imaging [24,29,35,40,104,105,106].

Features	PVS	Subcortical Infarct	Lacune	WMH	Giant PVS	Benign Intracranial Cysts	Neoplastic Intracranial Cysts	Vascular and Inflammatory Cysts	Infectious Cysts	Mucopolysaccharidosis
*FLAIR*	↓↓	↑↑	↓↓ with ↑ rim	↑↑	↓↓ with ↑ rim	↓↓ (↑ in neurenteric cysts)	↑↑with possible ↑ rim	↓↓ with ↑ rim	Variable	↓↓ with ↑ rim
*T2*	↑↑	↑↑	↑↑	↑	↑↑	↑↑	↑/↑↑	↑↑	Variable	↑↑
*T1*	↓↓	↓↓	↓↓	↓	↓↓	↓↓ (↑ in neurenteric cysts)	↓/↓↓	↓↓	Variable	↓↓
*Enhancement*	-	-	-	-	-	-	↑ of solid tissue	↑ in active inflammation	Variable (eccentric target sign for neurotoxoplasmosis, cyst wall for neurocysticercosis)	-
*DWI*	-	Restricted	-	-	-	Restricted in epidermoid cyst	Restricted	-	Restricted in cryptococcosis	-
*Size*	≤2 mm	≤20 mm	3–15 mm	≥3 mm	≥15 mm	≥15 mm	≥15 mm	≥3 mm	≥15 mm	≥3 mm
*Shape*	Dot-like or tubular	Dot-like or tubular	Wedge	Rounded or irregular	Bizarre cystic configurations	Rounded	Bizarre cystic configurations (“bubbly” for DNET)	Bizarre in encephalomalacia, linear or ovoid in multiple sclerosis	Rounded or irregular	Dot-like or tubular
*Location*	Symmetric in white matter or lower basal ganglia	Subcortical territory of a perforating arteriole	Asymmetric in white matter or upper basal ganglia	Symmetric in periventricular white matter	Mesencephalothalamic region (type III), anterior superior temporal lobe (type IV)	Single intra-axial, subarachnoid or intraventricular	Single intra-axial	Encephalomalacia have connections with the subarachnoid space; perivenular distribution in multiple sclerosis	Multiple intra-axials, subarachnoids or intraventriculars	Basal ganglia, corpus callosum, and white matter
*Other*	-	-	-	-	-	-	Change in follow up	Clinical picture	Clinical picture	Clinical picture

FLAIR: fluid attenuated inversion recovery; DWI: diffusion weighted imaging; WMH: white matter hyperintensities; DNET: dysembryoplastic neuroepithelial tumors; ↑ increased signal; ↓ decreased signal.

## Data Availability

Not applicable.
